# Retaking assessment system based on the inspiratory state of chest X-ray image

**DOI:** 10.1007/s12194-025-00888-0

**Published:** 2025-02-19

**Authors:** Naoki Matsubara, Atsushi Teramoto, Manabu Takei, Yoshihiro Kitoh, Satoshi Kawakami

**Affiliations:** 1 Division of Radiology, Shinshu University Hospital, 3-1-1 Asahi, Matsumoto, Nagano, 390-8621 Japan; 2 Faculty of Engineering, Meijo University, 1-501 Shiogamaguchi, Tempaku-ku, Nagoya, 468-8502 Japan; 3 Department of Radiology, Shinshu University School of Medicine, 3-1-1 Asahi, Matsumoto, 390-8621 Japan

**Keywords:** Chest X-ray, Retaking assessment, Inspiration state, Convolutional neural network, Dynamic digital radiography

## Abstract

When taking chest X-rays, the patient is encouraged to take maximum inspiration and the radiological technologist takes the images at the appropriate time. If the image is not taken at maximum inspiration, retaking of the image is required. However, there is variation in the judgment of whether retaking is necessary between the operators. Therefore, we considered that it might be possible to reduce variation in judgment by developing a retaking assessment system that evaluates whether retaking is necessary using a convolutional neural network (CNN). To train the CNN, the input chest X-ray image and the corresponding correct label indicating whether retaking is necessary are required. However, chest X-ray images cannot distinguish whether inspiration is sufficient and does not need to be retaken, or insufficient and retaking is required. Therefore, we generated input images and labels from dynamic digital radiography (DDR) and conducted the training. Verification using 18 dynamic chest X-ray cases (5400 images) and 48 actual chest X-ray cases (96 images) showed that the VGG16-based architecture achieved an assessment accuracy of 82.3% even for actual chest X-ray images. Therefore, if the proposed method is used in hospitals, it could possibly reduce the variability in judgment between operators.

## Introduction

### Background

**Fig. 1:**
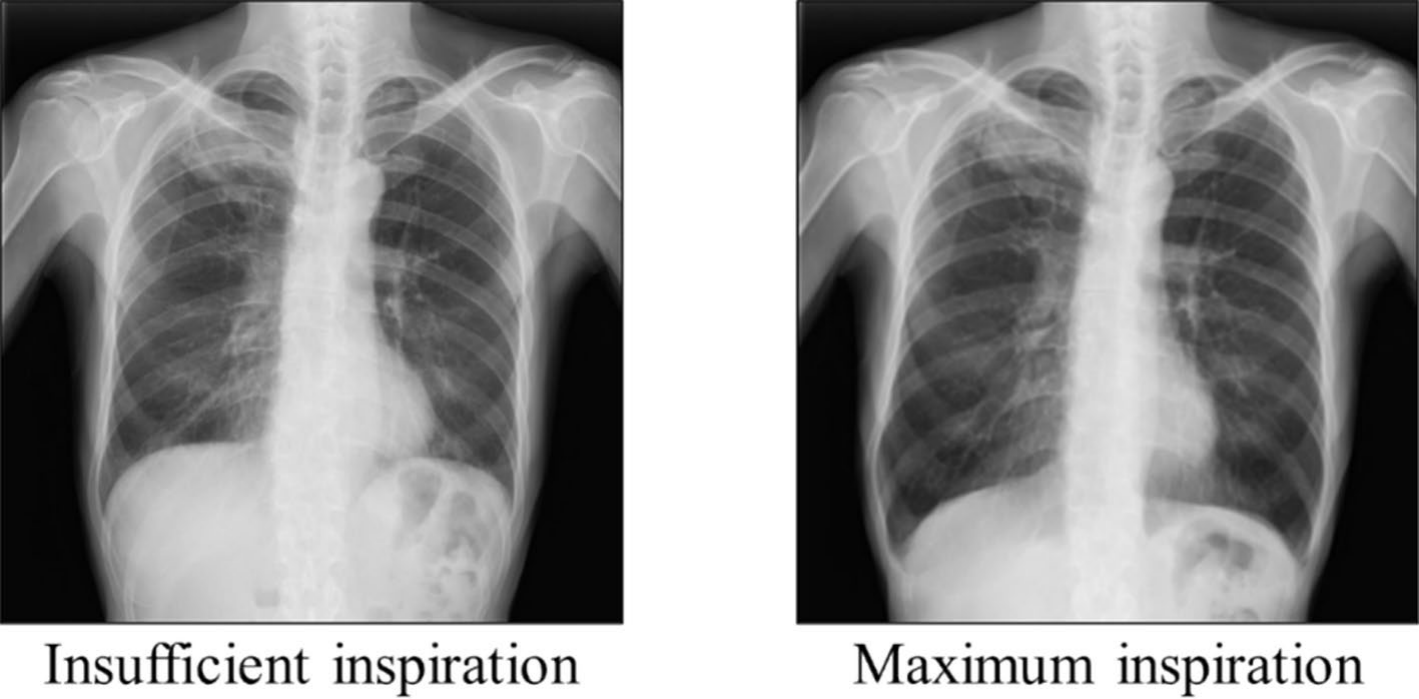
Chest X-ray images of pulmonary emphysema

Chest X-rays are often used for follow-up of patients with respiratory and circulatory diseases and for screening upon hospitalization. When taking chest X-rays, the patient is encouraged to take a maximum inspiration and the radiological technologist takes the images at the appropriate time. When physicians interpret images, the attention is often focused on factors such as lung volume, pulmonary permeability, and pulmonary vascular shadows [1]. Because these factors are interpreted on the assumption that the image was taken at maximum inspiration, images with insufficient inspiration may lead to an incorrect diagnosis. Figure 1 shows chest X-ray images of pulmonary emphysema. As shown in the maximum inspiration image on the right, hyperinflation of the lungs is an imaging finding of emphysema on chest X-ray images. However, this may be overlooked in the image of insufficient inspiration on the left, resulting in an incorrect diagnosis. Therefore, taking the image at maximum inspiration is extremely important. In addition, if the image is not captured at maximum inspiration, retaking the image is required. The decision as to whether retaking is necessary is usually made by a physician or radiological technologist immediately after taking the image, but it is thought that there is variation in the judgment between operators. This may be due to large differences in the amount of inspiration between patients and the fact that the judgment criteria differ depending on the operator.

Therefore, we considered that it might be possible to reduce variation in judgment using a computer to assess whether or not retaking was necessary, a decision that had previously been made at the discretion of the operator, and by providing the results to the operator as a second opinion. Furthermore, if the assessment is made with high accuracy, it may also lead to reduced medical exposure due to unnecessary retaking.

### Related works

Conventional computer-based image classification methods use developer-designed indexes and filters to calculate features from images and set criteria to obtain classification results. The advantage of this method is that the basis for classification is easy to understand and the disadvantage is that it requires a significant amount of effort and time to design the features. In this study, it was considered necessary to design features that could accommodate the physical differences between patients and that stable results could not be obtained with features calculated by simple image processing. However, methods using artificial intelligence, which have made significant progress in recent years, generate useful features from given data and make judgments, eliminating the need for developers to design the features. Furthermore, it has been reported that these methods often achieve higher processing accuracy than conventional methods [2]. In particular, convolutional neural networks (CNN) have demonstrated high accuracy in image recognition and many research cases have been reported that are directly related to radiology and the work of radiological technologists [3–10]. Matsubara et al. demonstrated that bone suppression in chest X-ray images can be processed with high accuracy using CNN as a spatial filter [3]. Tang et al. developed a system using CNN to distinguish between normal and abnormal chest X-ray images and demonstrated that it is processed accurately [4]. Rahman et al. automatically detected pneumonia and classified it as bacterial or viral with high accuracy by providing chest X-ray images to CNN [5]. Heidari et al. detected COVID-19 pneumonia with high accuracy by generating pseudo-color images using two different preprocessed images of chest X-ray images and using them as input data for CNN [6]. Keidar et al. proposed a tool that would allow COVID-19 patients from chest X-ray images using CNN and subsequently allow similar patients to be identified [7]. Nafisah et al. automatically detected tuberculosis with high accuracy by segmenting the lung field from chest X-ray images and providing it to CNN [8]. Ichikawa et al. showed that estimating a patient’s weight from chest X-ray images using CNN is useful for radiation dose management and determination of contrast medium dose [9]. In addition, a report on a retaking support system for knee joint X-ray examinations using CNN suggested that it improves the work efficiency of radiological technicians [10]. However, there has been no previous study on the retaking assessment system for chest X-ray images based on the inspiratory state.

### Purpose

The purpose of this study is to develop a retaking assessment system (RAS) that uses a CNN to assess the need for retaking chest X-rays by focusing on the inspiration state. This could allow doctors to receive diagnostically appropriate images taken at maximum inspiration without being affected by the patient’s physique or operator skill.

## Methods

### Overview

**Fig. 2:**
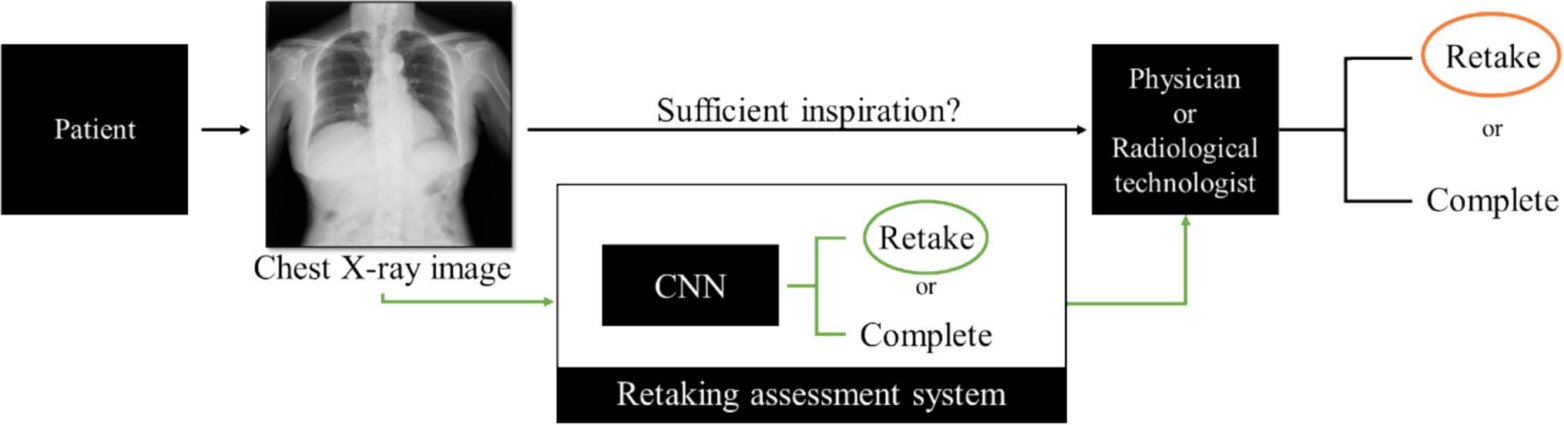
Overview of proposed flow

An overview of the proposed method is shown in Fig. 2. The chest X-ray image taken is immediately given to the RAS, which outputs an assessment of whether retaking is required or not. The assessment result was provided to the operator as a second opinion, after which the operator made a final decision.

For the CNN to provide whether retaking is necessary, training using the input data and corresponding labels is required. In this study, the input data were chest X-ray images and labels indicated whether retaking was required. However, chest X-ray images alone cannot distinguish whether inspiration is sufficient and retaking is unnecessary or insufficient and retaking is required. Therefore, we generated input images and labels from dynamic digital radiography (DDR) and used them to train and validate a CNN [11, 12]. In addition, we verified the effectiveness of the proposed method using actual chest X-ray images. The details of this process are provided below.

### Dataset

#### Dynamic digital radiographs (DDR)

This study included dynamic digital radiographs of 80 cases examined at Shinshu University Hospital between October 14, 2020, and April 18, 2022. Of these, 18 cases remained after excluding cases taken after surgery, such as lung resection and cases in which drains were inserted. DDR was conducted using an Aero DR fine (KONICA MINOLTA, Tokyo, Japan) flat panel detector and a UD150B-40 (SHIMAZU CORPORATION, Kyoto, Japan) X-ray system. The images were automatically analyzed using the workstation KINOSIS (KONICA MINOLTA, Tokyo, Japan). The matrix size was 1062 × 1062 pixels.

**Fig. 3:**
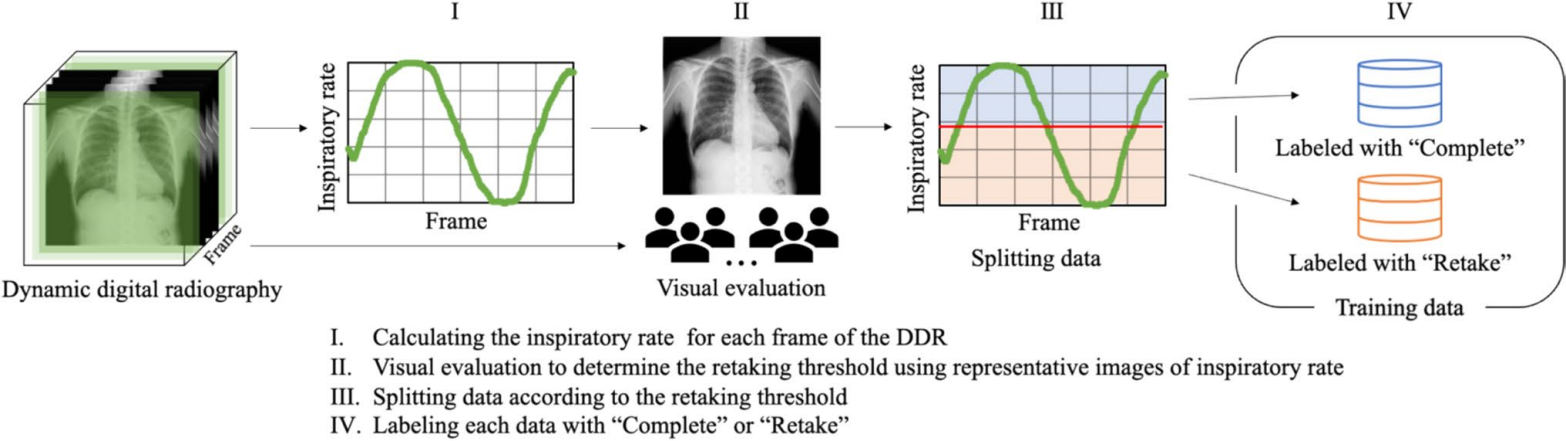
The flow of training data preparation

Figure 3 shows the flow of preparing training data from the DDR. When taking the DDR, the patient was instructed by an automatic voice to breathe in the following order: maximum inspiration → maximum expiration → maximum inspiration. The images were taken continuously at 15 frames per second and 300 frames were obtained in one examination. By providing the acquired images to a workstation, the upper and lower edges of the lung field were automatically detected for each frame and the position data of these two points was obtained. The relative distance between the two points was calculated using Eq. (1), and the calculated value was defined as the inspiration rate for each frame. In addition, the images were converted to the PNG format with a matrix size of 224 × 224 pixels by bicubic interpolation.

1 $${\text{Inspiratory rate}}\left( i \right) = \frac{{D\left( i \right) - D_{{{\text{min}}}} }}{{D_{{{\text{max}}}} - D_{{{\text{min}}}} }} \times 100$$

where *D*(*i*) is the distance D between the upper and lower ends of the lung field in frame *i*, *D*_max_ is the maximum distance *D* during the examination, and *D*_min_ is the minimum distance.

Visual evaluation was performed to set the retaking threshold based on the inspiration rate. For this evaluation, we used RadiForce RX360 (EIZO Corporation, Ishikawa, Japan), a 3-megapixel (1536 × 2048 pixels) liquid–crystal display (LCD). The LCD was calibrated based on the grayscale standard display function described in Digital Imaging and Communications in Medicine Part 14 [13], with a recommended luminance of 500 cd/m^2^. The illuminance of the observation environment complied with the JIS Z 9110 [14]. The observers included 10 radiological technologists (10.4 ± 6.6 years of clinical experience). Informed consent was obtained from all observers to participate in the study and disclose the results of their visual evaluations. In addition, we obtained agreement to cancel the evaluation results if participants expressed a desire to withdraw from the study. The observers who participated in this study routinely took chest X-ray images. We deliberately did not provide prior training to ensure that clinical judgment was as accurately reflected as possible in the visual assessments. In total, 108 images with inspiratory rates of 0, 20, 40, 60, 80, and 100% were used in each case. With regard to visual evaluation methods for creating training data, some reports reflect the results of one or two observers [15–17]. In addition, other studies incorporate the evaluation results of a third observer only when the results of two observers diverged [18], made a final decision through consensus when the results of two observers contradicted each other [19] or used most of the evaluation results from three observers as the ground truth [4]. Based on these studies, we adopted the 2-phase visual evaluation method to generate reliable training data.

**Fig. 4:**
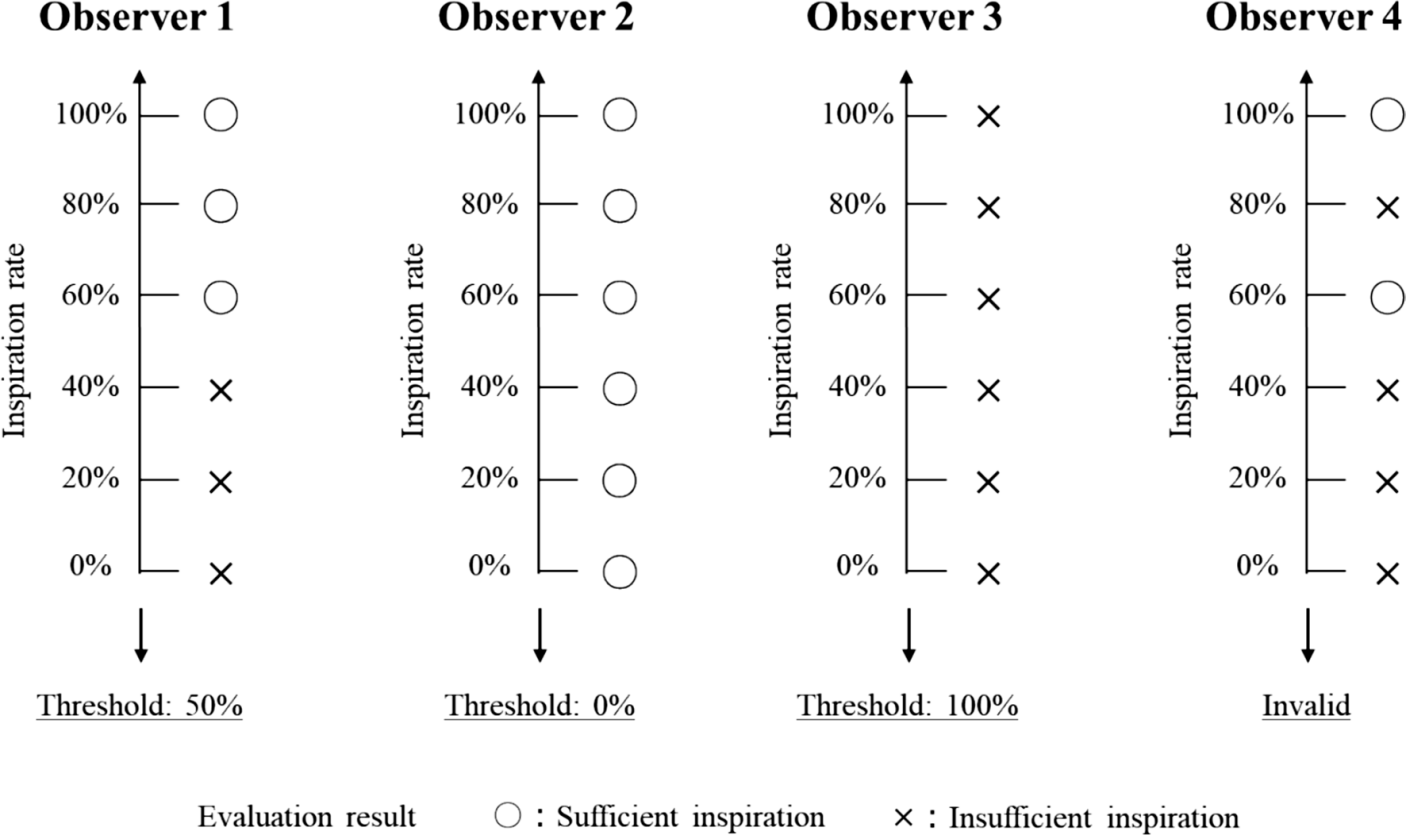
Analysis method of visual evaluation results

In phase 1, all the images were randomly sorted and displayed individually. Each radiological technologist conducted evaluations independently, with two options: “Inspiration is sufficient” or “Inspiration is insufficient.” The observation time was set at approximately 3 s per image to reflect the clinical work situation. In addition, re-evaluating an image once it had been rated was prohibited, and the evaluation was based on only one displayed image. The evaluation results were analyzed for each case and observer, and the threshold of individual observer was obtained. An example of this is shown in Fig. 4. If the evaluationresults were divided at a certain inspiratory rate, that rate was set as the threshold (Observer 1). If the same evaluation result was obtained for all inspiratory rates, the threshold was 0% or 100% (Observer 2 or 3). If the evaluation results were mixed, they were excluded from the analysis (Observer 4). In addition, the mean and standard deviation (S.D.) of the threshold of individual observer were calculated. If the change in the images was small despite the different inspiratory rates, the evaluation results would likely be “invalid.”

In phase 2, the consensus threshold was determined through a consensus between the 10 radiological technologists by reviewing the results of phase 1 and images of each case. As a result of phase 1, the mean and S.D. of the threshold of individual observer, and the number of invalid results were displayed. The images presented were the same as those in phase 1 (inspiratory rates of 0, 20, 40, 60, 80, and 100%). For comparison at a glance, the images were arranged in descending order of inspiratory rates for each case. The observers were the same as those in phase 1, and the time for discussion was arbitrary. The consensus threshold was selected from inspiratory rates of 0, 10, 30, 50, 70, 90, and 100%. Table 1 presents the results of the study. The results of each observer in Table 1 (threshold of individual observer) are the reference data, and the threshold on the far right (consensus threshold) was used as the retaking threshold for training.

**Table 1:** Results of visual evaluation

Case number	The threshold of individual observer (Phase 1)												Consensus threshold (Phase 2)
	A	B	C	D	E	F	G	H	I	J	Mean	S.D	
001	100	100	90	90	–	100	70	70	–	90	88.8	11.7	70
002	0	0	0	0	0	0	0	–	10	0	1.1	3.1	30
003	30	–	30	50	10	10	30	–	30	30	27.5	12.0	50
004	-	50	–	50	90	50	50	50	–	50	55.7	14.0	70
006	90	90	100	–	90	70	–	50	90	70	81.3	15.4	70
007	0	10	–	50	0	–	–	50	–	–	22.0	23.2	50
008	50	70	70	50	-	90	50	50	100	70	66.7	17.6	70
009	0	0	70	0	0	30	10	0	0	0	11.0	21.7	50
011	50	50	90	70	30	70	50	50	50	50	56.0	15.6	50
013	10	0	–	–	–	30	–	10	0	10	10.0	10.0	50
015	90	100	90	90	90	100	–	90	100	90	93.3	4.7	90
016	70	-	10	70	10	50	50	50	70	70	50.0	23.1	70
018	-	30	30	30	50	30	–	–	70	30	38.6	14.6	70
019	30	50	70	70	100	30	30	30	50	70	53.0	22.8	90
021	90	–	70	70	100	50	10	10	50	–	56.3	31.2	70
025	70	70	–	70	70	70	90	50	100	90	75.6	14.2	70
026	-	–	50	70	100	30	30	–	–	70	58.3	24.8	50
027	10	–	–	0	0	10	10	10	70	30	17.5	21.7	70

The threshold of individual observer, its mean, and S.D. were obtained by visual evaluation phase 1, while the consensus threshold was obtained in phase 2 and used as the retaking threshold for training. "-" indicates invalid

Frames with an inspiratory rate higher than the retaking threshold were labeled as a complete examination (“Complete”), whereas frames with an inspiratory rate equal to or lower than the retaking threshold were labeled as requiring retaking (“Retake”). The 300 frames obtained from one DDR examination were added to the CNN training data as 300 chest X-ray images.

In addition, a questionnaire was conducted to clarify the areas that the observers paid attention to when checking the state of inhalation on chest X-ray images. This questionnaire was conducted immediately after the visual assessment, and the response time was arbitrary. The options included the position of the upper end of the diaphragm and costophrenic angle, radiolucency within the lung fields, expansion of the thorax, overlapping diaphragm and cardiac shadows, cardiac shadow, the position of the clavicle, and others. Theparticipants were allowed to select all applicable options. The result of the questionnaire is shown in Table 2.

**Table 2:** Results of the questionnaire regarding the area to pay attention to when checking the inspiratory state

	Number of observers (ratio)
Position of the upper end of diaphragm	7 (70%)
Position of costophrenic angle	7 (70%)
Radiolucency within the lung fields	7 (70%)
Expansion of the thorax	6 (60%)
Overlapping diaphragm and cardiac shadows	5 (50%)
Cardiac shadow	1 (10%)
Position of the clavicle	1 (10%)
Other	0 (0%)

#### Chest X-ray images

CNN was trained using DDR, whereas our proposed method targets actual chest X-ray images. Therefore, we conducted an additional validation to confirm whether the proposed method is useful for actual chest X-ray images. This study involved 95 chest X-ray cases in which patients were requested to take two images, one in the maximum inspiration state and one in the maximum expiration state, at Shinshu University Hospital between October 14, 2020, and December 1, 2023. Patients whose images were taken in a position other than standing and those that overlapped with chest dynamic digital radiographs were excluded. Consequently, 48 patients were included (96 images). These images were obtained using three different X-ray machines. Twelve cases were obtained using a Digital Diagnosis C90 (Philips Healthcare, Cleveland, OH, USA), 35 cases were obtained using a UD150B-40 (SHIMAZU) X-ray generator and CXDI-401C (CANON Medical Systems, Otawara, Japan) flat panel detector, and one case was obtained using a UD150B-40 (SHIMAZU CORPORATION, Kyoto, Japan) X-ray generator and an Aero DR fine (KONICA MINOLTA, Tokyo, Japan) flat panel detector. The maximum matrix size was 3320 × 3408 pixels.

**Fig. 5:**
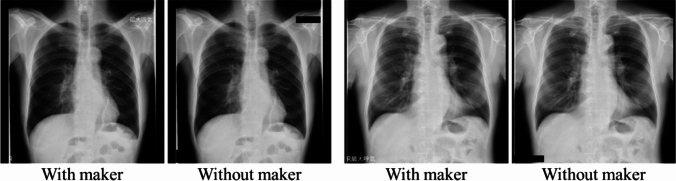
The example of removing the image marker

The markers in the images were removed. The target area was specified and the pixel value was replaced with zero using ImageJ [20]. TheExamples are shown in Fig. 5. Furthermore, the image format was changed to PNG and the matrix size was converted to 224 $\times$ 224 pixels using bicubic interpolation.

### Image classification

As mentioned previously, we used a CNN to assess whether retaking was necessary. Transfer learning was introduced to train the CNN. Transfer learning is a technique that uses weights learned for one task as a starting point for another task and has the advantage of being efficiently trained even with small datasets [21]. In this method, the weights trained on ImageNet [22] were used as the initial values and the fully connected layers and beyond were additionally trained using the DDR. Here, the X-ray image is a one-channel grayscale image, whereas the input information for the CNN is a color image. Therefore, we assigned the same grayscale image to each plane of the three channels of the color image and fed it into the CNN. When a chest X-ray image is provided to the trained CNN, it outputs whether or not retaking is necessary.

### Evaluation method

#### Cross-validation using DDR

To confirm the effectiveness of the proposed method, we conducted a six-fold cross-validation using DDR. Of the 18 dynamic digital radiographs, 13 were used as training data, two as validation data, and three as test data. The validation was conducted six times, rotating the cases so that each case served as test data. As shown in 2.2.1, one DDR consists of 300 frames. Each frame was used as a normal chest X-ray image for verification. In addition, the training data were augmented using image enlargement processing to address differences in body size. Two enlargement ratios (× 1.045 and × 1.090) were applied to the “Complete” images to triple the amount of data, and three enlargement ratios (× 1.03, × 1.06, and × 1.09) were applied to the “Retake” images to increase the amount of data four-fold. Consequently, the number of training data was approximately 13,500 images per fold, with an improved balance between the “Complete” and “Retake” categories.

**Fig. 6:**
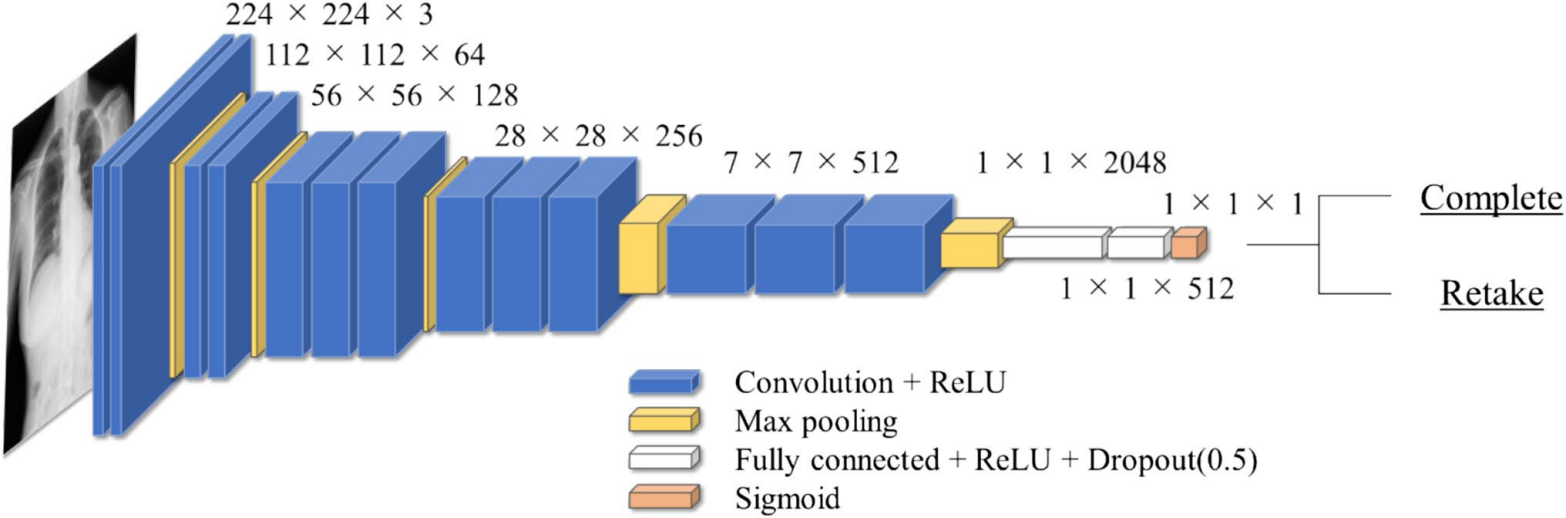
CNN architecture based on VGG16

Seven well-known CNN architectures namely VGG16, VGG19 [23], InceptionV3 [24], ResNet50 [25], DenseNet121, DenseNet169, and DenseNet201 [26], were adopted to compare processing accuracy and determine the most suitable model for the proposed method. In addition, to adapt each model to the target task, the fully connected layer was modified to assess whether retaking was necessary. The fully connected layer had a three-layer structure with each layer having 2048, 512, and 1 units. The Sigmoid function was used for the final layer. The VGG16-based architecture is shown in Fig. 6. The batch size was set to 128, and Adam (learning rate (Lr) = 1e−4, 1e−5, and 1e−6) was used as the optimization function [27]. Binary cross-entropy was used as the loss function. Early stopping was adopted to avoid overfitting and the maximum number of epochs was set to 100. The validation function was monitored and training was stopped if no improvement was observed within three iterations. The hardware used for calculations were an Intel Core i9-12900 CPU(Intel, Santa Clara, CA, USA) and NVIDIA GeForce 3090 GPU(Intel, Santa Clara, CA, USA) with TensorFlow (Google, Mountain View, USA) and Keras software. Furthermore, a heat map was generated using Grad-CAM [28] to visualize the areas of the image CNN focused on for assessment.

#### Verification using actual chest X-ray images

Verification was performed using chest X-ray images to confirm the usefulness of the proposed method. All DDR cases were used for training and randomly divided into 15 (4500 images) and three (900 images) cases for the training and validation data, respectively. The test data consisted of 96 actual chest X-ray images. The system was retrained using a larger dataset than that used in the cross validation to process the actual chest X-ray images.

The three architectures demonstrated high accuracies in the verification described in Sect. 2.4.1 were adopted as the CNN models. The hardware, software, and parameters were the same as those described in the previous section. A heat map was generated using Grad-CAM in the same manner.

## Results

### Six-fold cross-validation using DDR

**Fig. 7:**
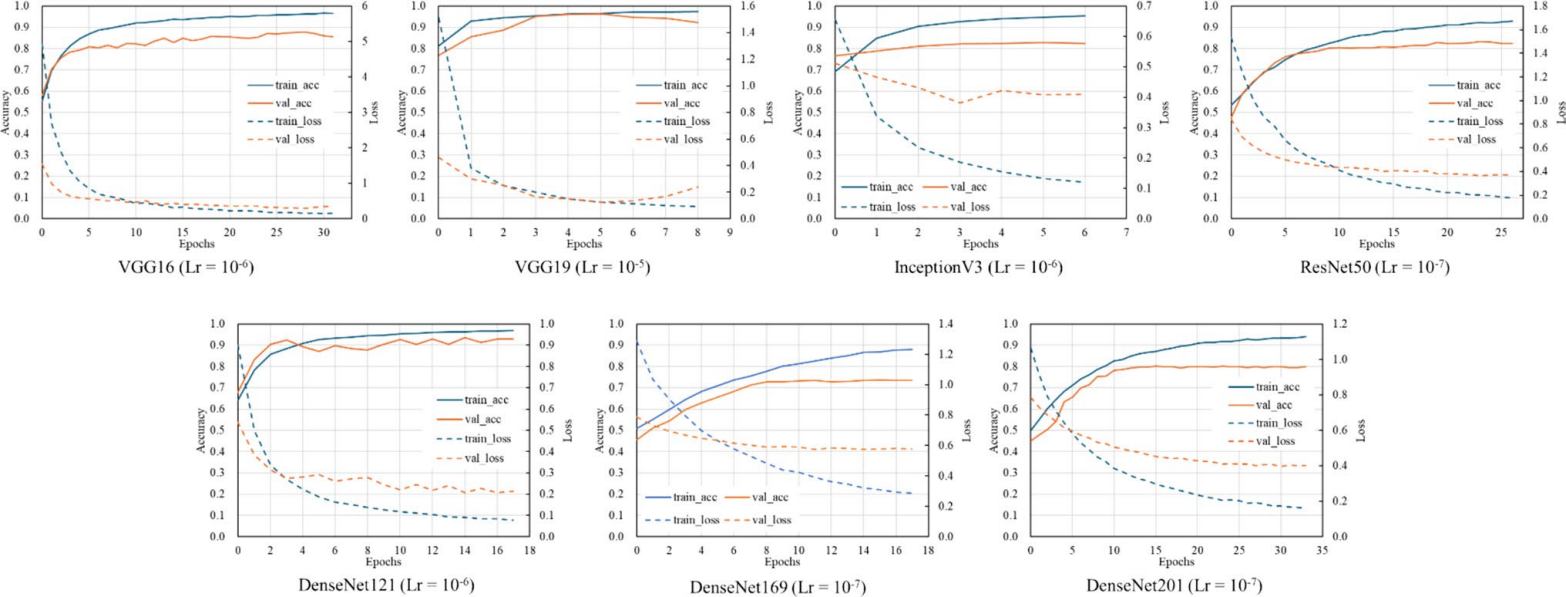
Examples of learning curves for each architecture. The horizontal axis indicates the number of epochs, and the vertical axis indicates accuracy and loss

**Fig. 8:**
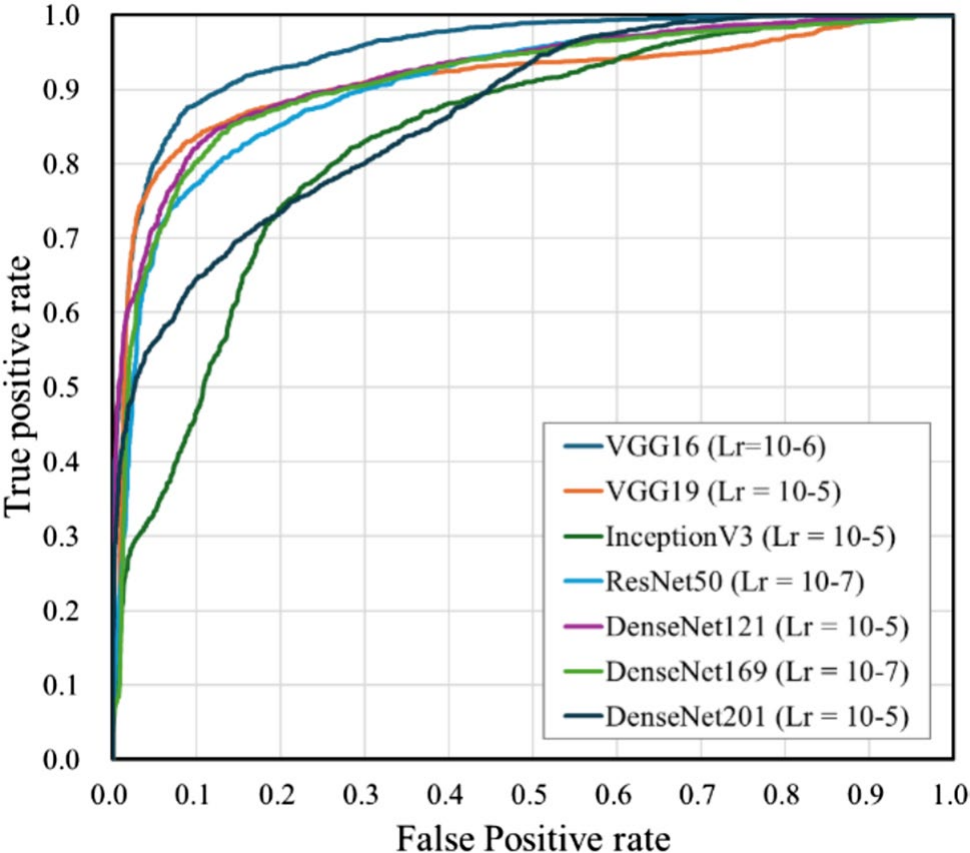
Receiver operating characteristic curves for each architecture

**Fig. 9:**
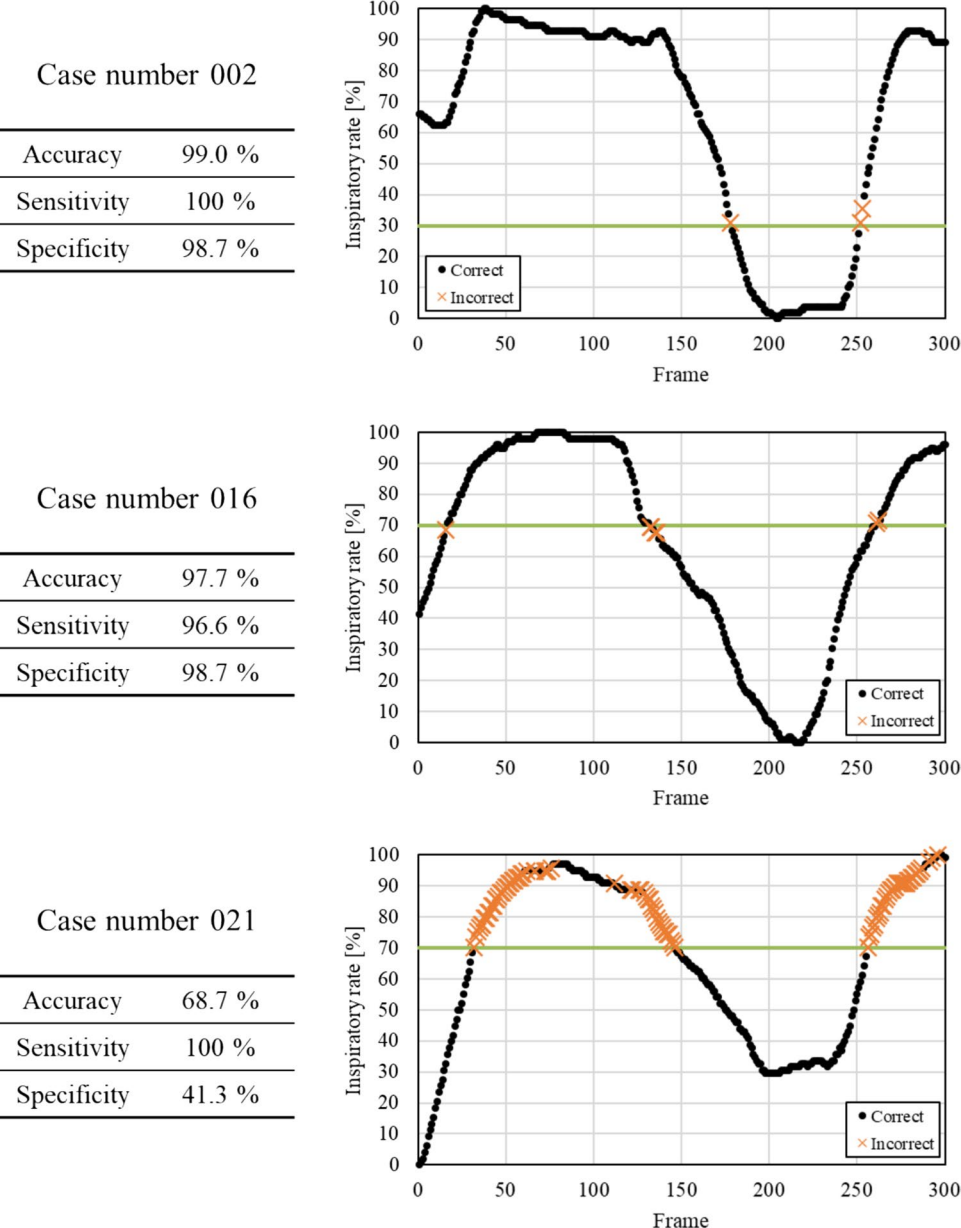
Detailed results of typical assessed cases

**Fig. 10:**
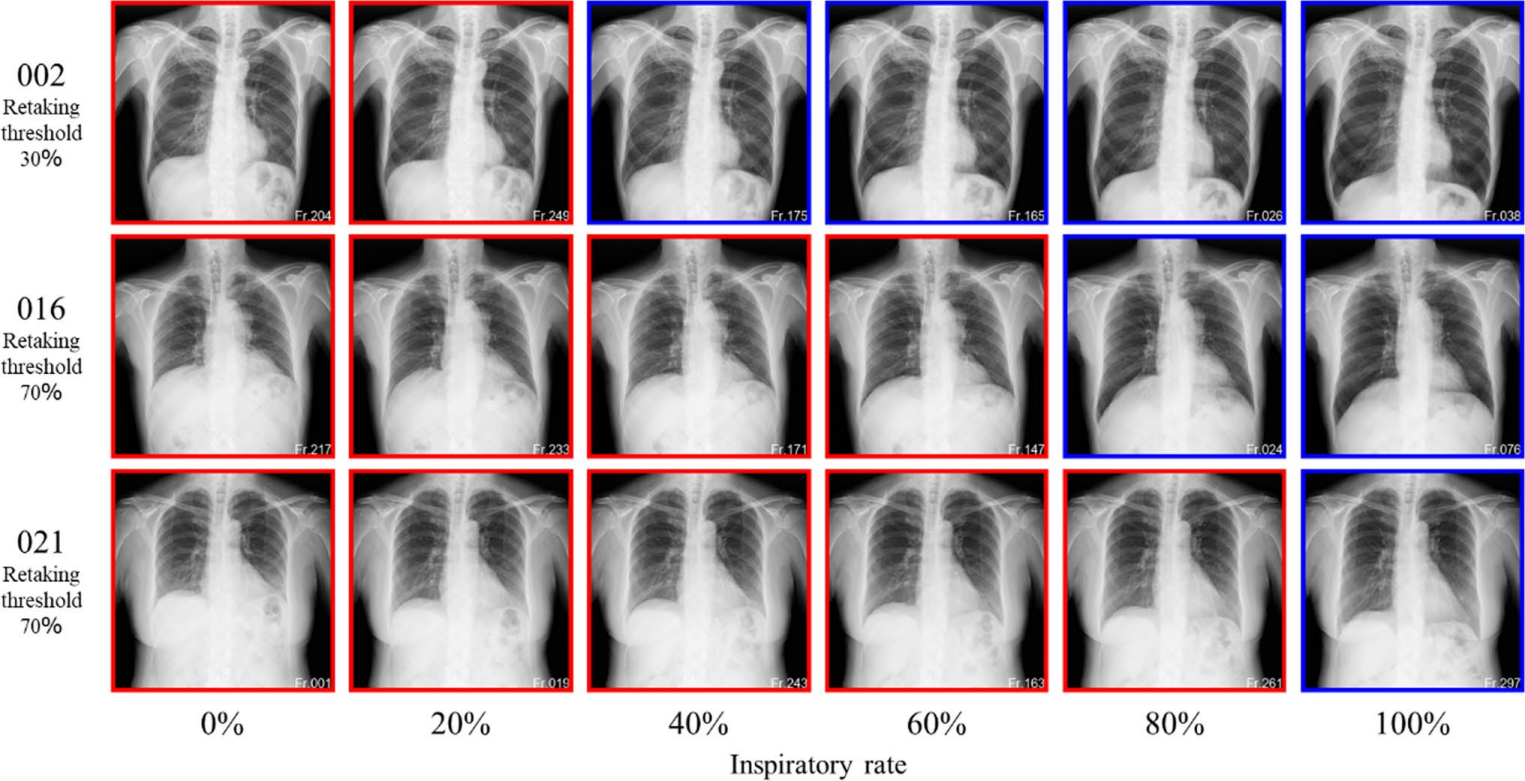
Assessment results for representative inspiratory rate images. The color of the outer border of the image indicates whether the radiograph was assessed as requiring retaking. The red line indicates that the image was assessed as “Retake,” and the blue line indicates that the image was assessed as “Complete.”

**Fig. 11:**
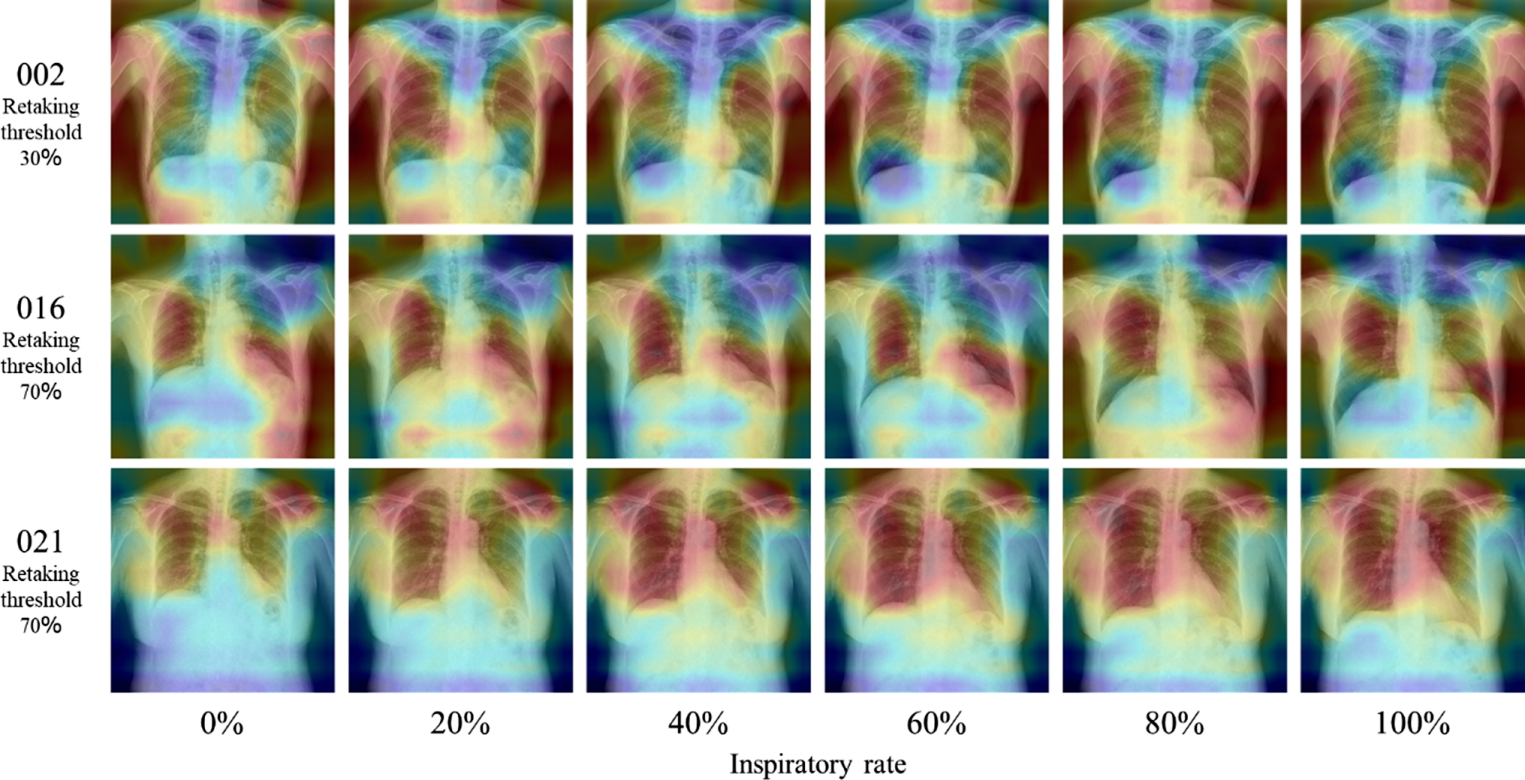
The analysis results by Grad-CAM

Table 3 shows the results of comparing the assessment accuracies of the different architectures. The highest accuracy of 89.5% was achieved by introducing a VGG16-based architecture (Lr = 10^–6^). Figure 7 shows the examples of learning curve for the learning rates that achieved the highest accuracy of each model. The average number of epochs was 14.5 for VGG16, 7.8 for VGG19, 8.7 for InceptionV3, 19.5 for ResNet50, 31.7 for DenseNet121, 17.7 for DenseNet169, and 25.7 for DenseNet201. VGG16 (Lr = 10^–6^) also achieved the highest precision, recall, F1 score, and AUC values. The receiver operating characteristic (ROC) curves for the highest AUC for each model are shown in Fig. 8. The assessment accuracies for each case using VGG16 (Lr = 10^–6^) are shown in Table 4, which shows that 11 cases were assessed with an extremely high accuracy of over 90%. In contrast, a case was assessed with an accuracy of 80% or less. The detailed assessment results for each case are shown in Figs. 9 and 10. The graph on the right side of Fig. 9 shows the inspiratory rate of each frame and whether the CNN assessment results are correct. The green line indicates the retaking threshold. If an × is marked below the retaking threshold, it indicates that the image that needs to be retaken was incorrectly assessed as “Complete.” In addition, in Fig. 10, the color of the outer border of the image indicates the assessment results of the CNN. The red line indicates that the image was assessed as “Retaking,” and the blue line indicates that the image was assessed as “Complete.” The results of the Grad-CAM analysis are shown in Fig. 11.

**Table 3:** Comparison of assessment accuracy of different architectures

	Learning rate	Accuracy	Precision	Recall	F1 score	AUC
VGG16	10^–5^	0.880	0.879	0.841	0.860	0.925
	10^–6^	0.895	0.886	0.873	0.879	0.955
	10^–7^	0.870	0.839	0.868	0.853	0.949
VGG19	10^–5^	0.877	0.876	0.835	0.855	0.923
	10^–6^	0.861	0.831	0.856	0.844	0.913
	10^–7^	0.815	0.769	0.822	0.795	0.890
InceptionV3	10^–5^	0.766	0.704	0.800	0.749	0.835
	10^–6^	0.784	0.723	0.818	0.768	0.834
	10^–7^	0.754	0.681	0.821	0.745	0.819
ResNet50	10^–5^	0.839	0.838	0.782	0.809	0.906
	10^–6^	0.826	0.786	0.826	0.805	0.894
	10^–7^	0.844	0.847	0.784	0.814	0.907
DenseNet121	10^–5^	0.865	0.879	0.801	0.838	0.923
	10^–6^	0.869	0.864	0.832	0.848	0.907
	10^–7^	0.850	0.853	0.793	0.822	0.922
DenseNet169	10^–5^	0.847	0.850	0.789	0.818	0.887
	10^–6^	0.818	0.799	0.780	0.789	0.877
	10^–7^	0.858	0.865	0.799	0.831	0.912
DenseNet201	10^–5^	0.775	0.751	0.726	0.738	0.869
	10^–6^	0.782	0.740	0.773	0.756	0.851
	10^–7^	0.793	0.743	0.802	0.772	0.859

**Table 4:** Details of assessment results by VGG16 (for each case)

Case number	1	2	3	4	6	7	8	9	11
Accuracy [%]	88.7	99.0	96.7	86.0	93.0	92.0	93.0	86.0	90.3
Case number	13	15	16	18	19	21	25	26	27
Accuracy [%]	98.3	82.0	97.7	80.3	81.3	68.7	94.0	90.0	94.7

### Verification using actual chest X-ray images

**Fig.12:**
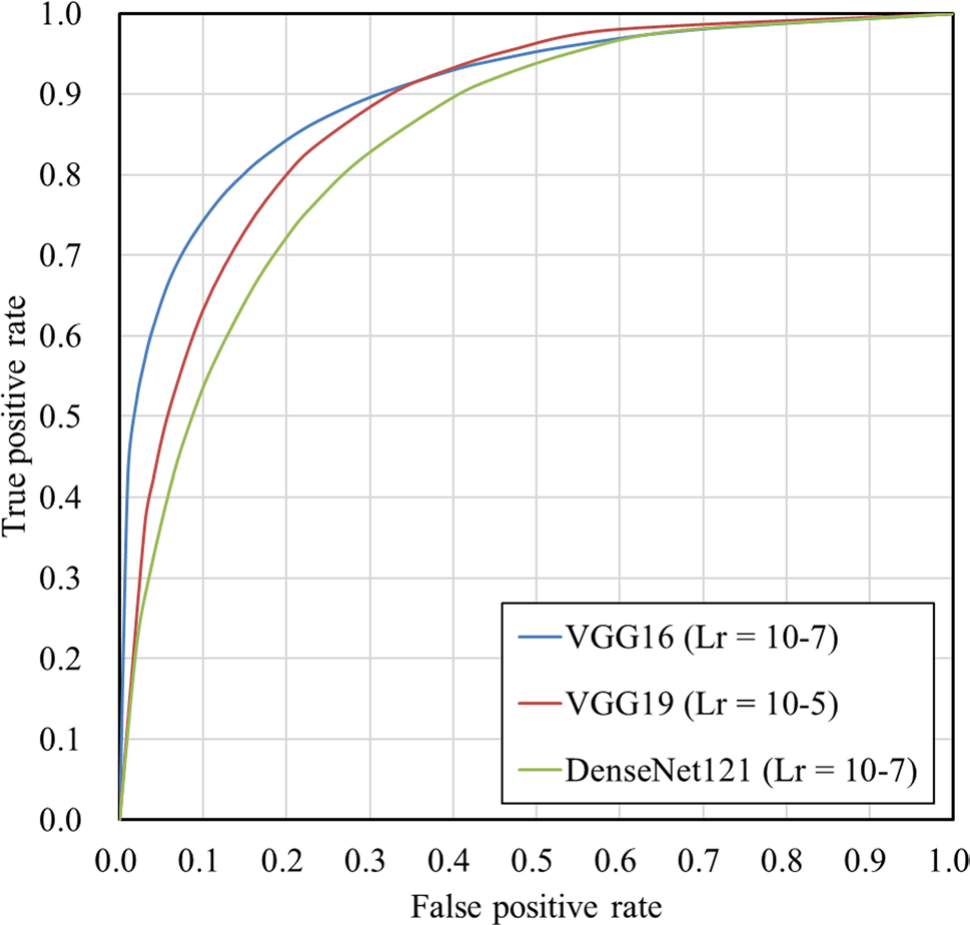
Receiver operating characteristic curves for each architecture

**Fig. 13:**
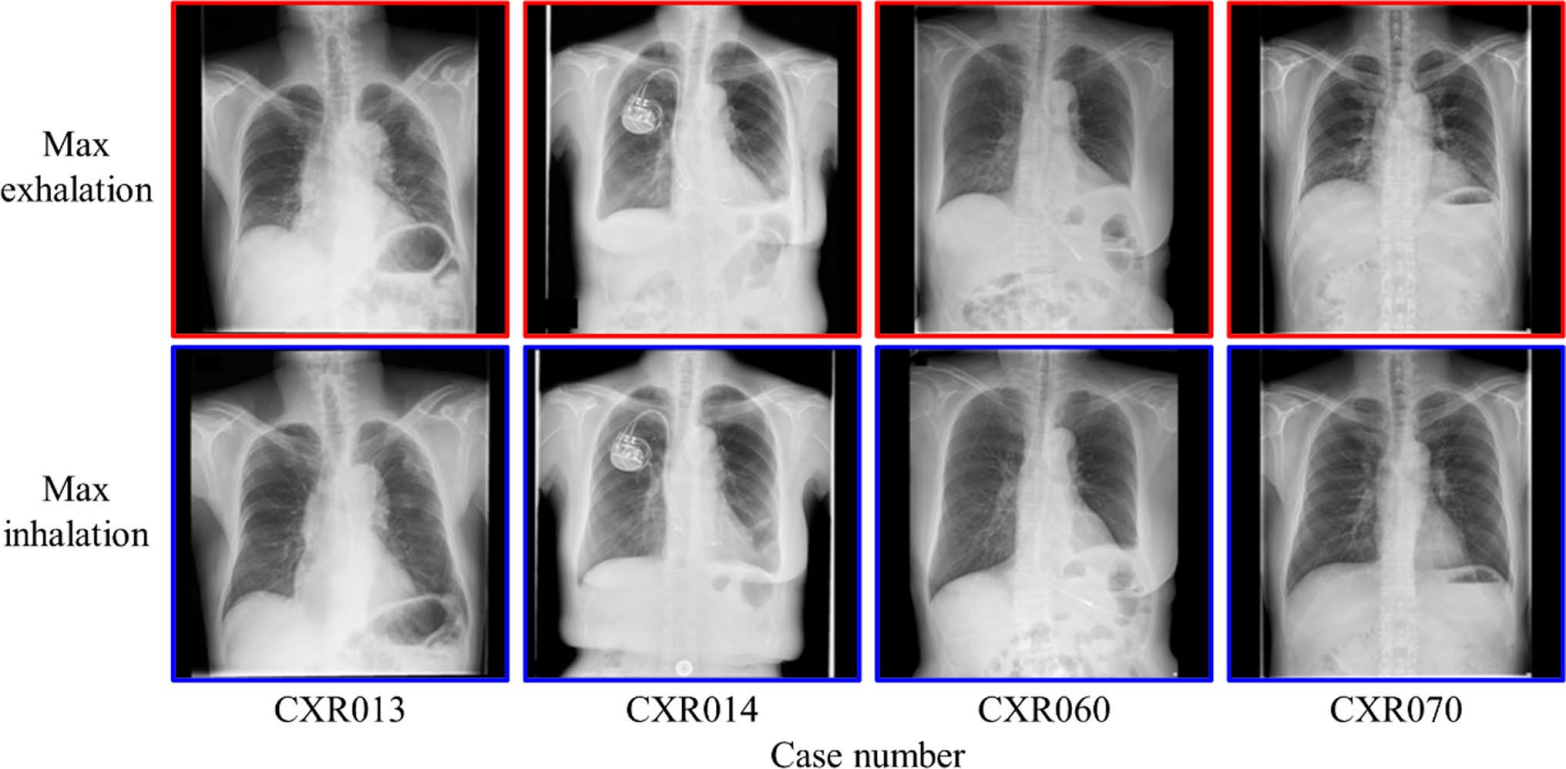
Examples of cases in which both maximum inhalation and exhalation images were correctly assessed. The color of the outer border of the image indicates whether the image was assessed as requiring retaking. The red line indicates that the image was labeled as “Retake,” and the blue line indicates that the image was labeled as “Complete.”

**Fig. 14:**
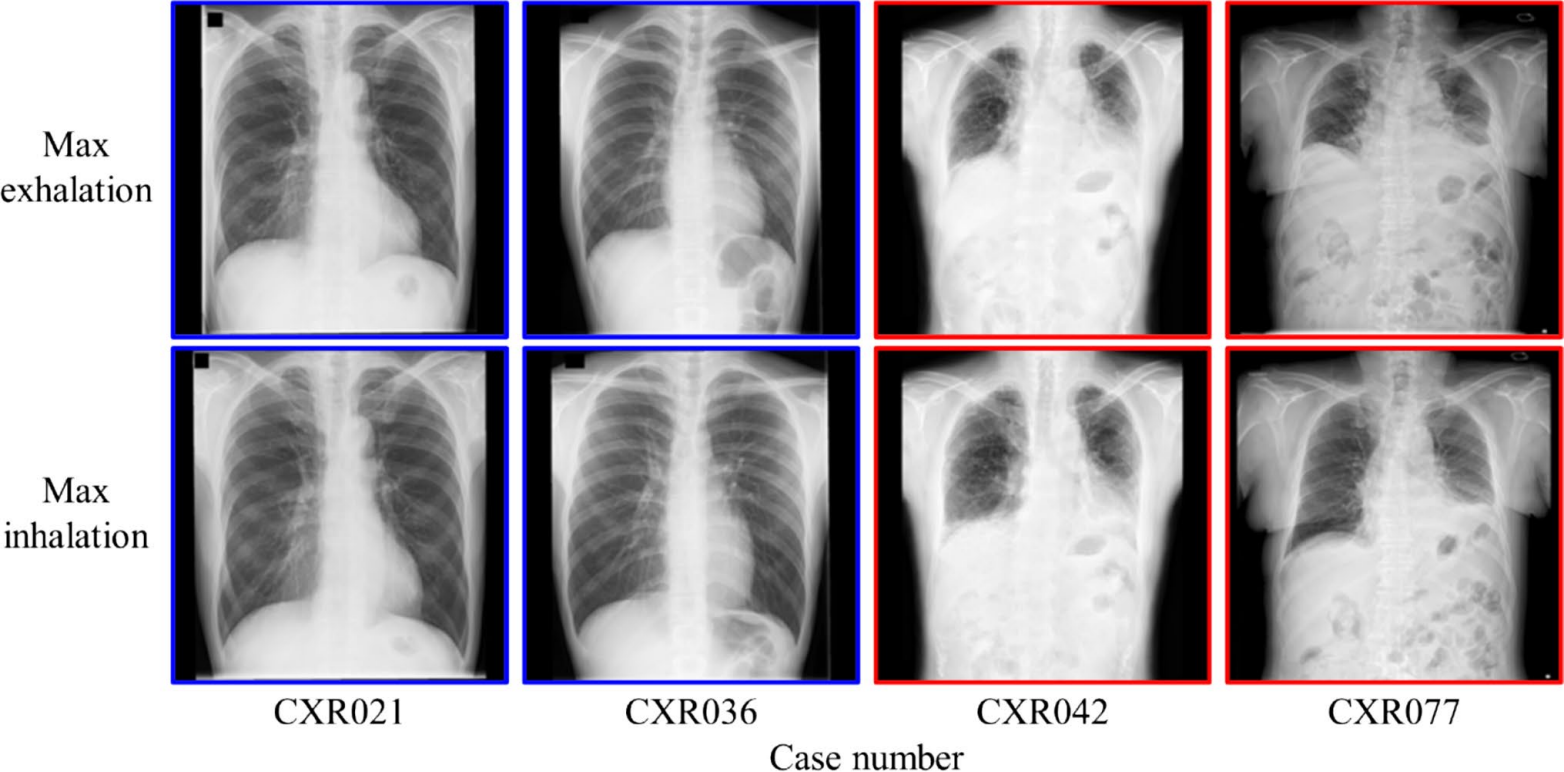
Example of cases in which one of the images was evaluated correctly. The color of the outer border of the image indicates whether it was assessed as requiring retaking. The red line indicates that the image was labeled as “Retake,” and the blue line indicates that the image was labeled “Complete.”

**Fig. 15:**
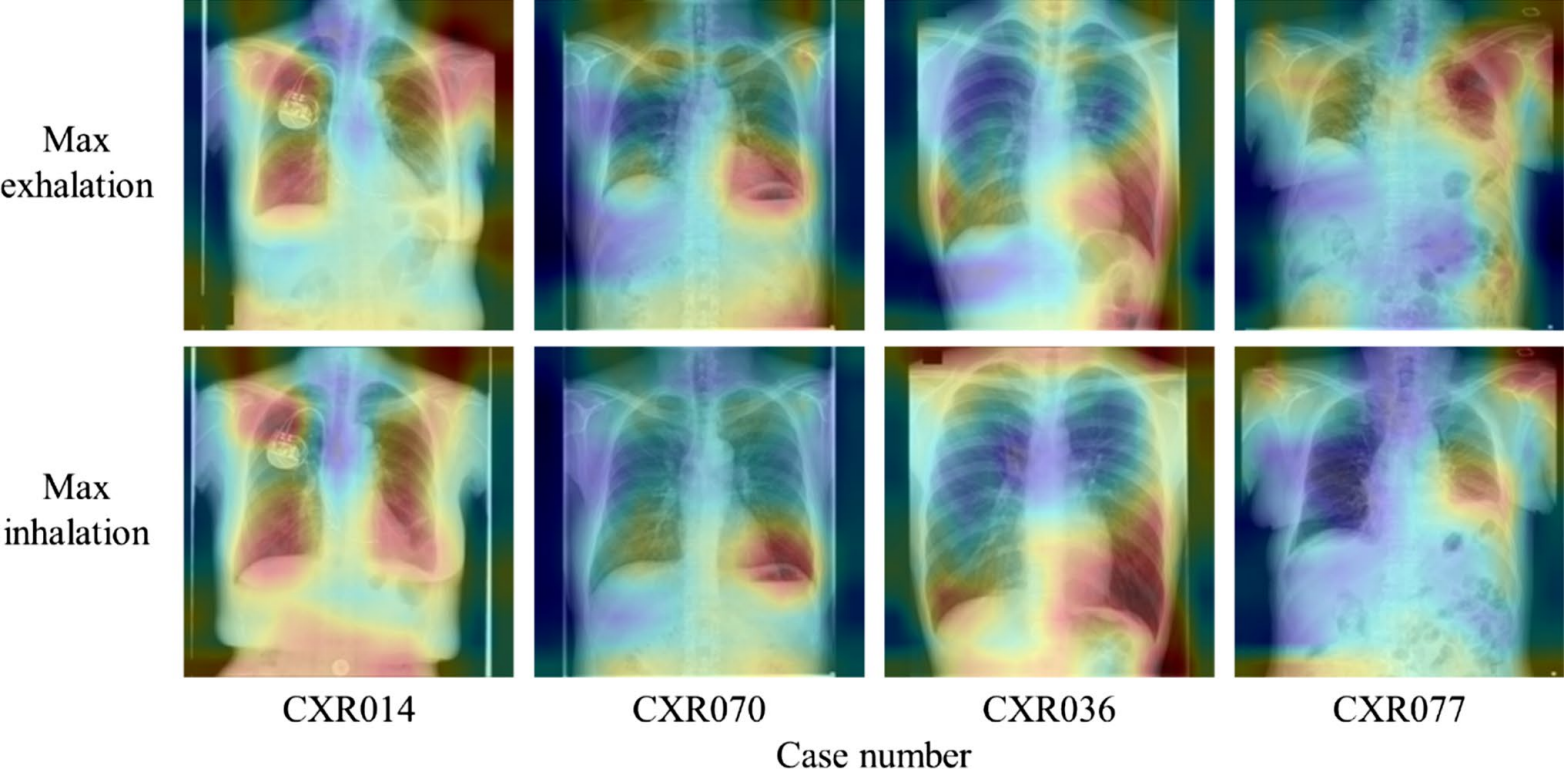
The analysis results by Grad-CAM

As shown in the previous section, architectures based on VGG16, VGG19, and DenseNet121 demonstrated higher accuracy. Therefore, in this verification, we used these architectures to verify the assessment accuracy on actual chest X-ray images. The results are summarized in Table 5. The highest assessment accuracy of 82.3% was achieved by VGG16 (Lr = 10^–7^), which also yielded the highest values for precision, F1 score, and AUC. The ROC curves of the highest AUC for each model are shown in Fig. 12. Table 6 presents the details of the judgment results obtained by VGG16 (Lr = 10^–7^) and the number of cases requested by the respiratory medicine department. Table 6 shows no cases in which both the maximum inhalation and exhalation images were incorrectly assessed, and 31 cases in which both were correctly assessed. In addition, 7 of the 10 cases that were correctly assessed for exhalation images only were requested by the Department of Pulmonary Medicine. Examples of the correctly and incorrectly assessed cases are shown in Figs. 13 and 14, respectively. The color of the outer border of the image indicates whether the assessment concluded that retaking was necessary. The red line indicates that the image was assessed as “Retake,” and the blue line indicates that the image was assessed as “Complete.” Fig. 15 shows the results of the analysis using Grad-CAM.

**Table 5:** Comparison of assessment accuracy of different architectures

	Learning rate	Accuracy	Precision	Recall	F1 score	AUC
VGG16	10^–5^	0.750	0.700	0.875	0.778	0.875
	10^–6^	0.792	0.750	0.875	0.808	0.832
	10^–7^	0.823	0.804	0.854	0.828	0.888
VGG19	10^–5^	0.792	0.741	0.896	0.811	0.862
	10^–6^	0.781	0.729	0.896	0.804	0.835
	10^–7^	0.771	0.710	0.917	0.800	0.851
DenseNet121	10^–5^	0.760	0.745	0.792	0.768	0.823
	10^–6^	0.760	0.755	0.771	0.763	0.812
	10^–7^	0.771	0.741	0.833	0.784	0.828

**Table 6:** Details of assessment results by VGG16 (Lr = 10^–7^) for each case (maximum exhalation and inhalation image)

Assessment result	Examination request from department		Total
	pulmonary medicine	Other	
Both are correct	18	13	31
Correct answer for max exhalation image	7	3	10
Correct answer for max inhalation image	4	3	7
Both are incorrect	0	0	0

## Discussion

As presented in Table 3, verification using DDR showed that the VGG16-based model (Lr = 10–6) achieved the highest value in all analysis metrics (accuracy, precision, recall, F1 score, and AUC) among the CNN architectures used in this study. In particular, the recall of 0.873 indicates that images with insufficient inspiration were correctly assessed as “Retake” with a high proportion. This prevents images with insufficient inspiration from being sent to the physician and it is believed that retaking will enable the provision of images suitable for diagnosis. Tang et al. and Keider et al. reported classification systems for detecting masses, nodules, and pneumonia on chest X-ray images. They demonstrated that although VGG16 achieved a certain level of accuracy, it was inferior to other models [4, 7]. In contrast, in a study by Usman et al. evaluating the presence of pneumonia in pediatric chest X-ray images, VGG16 demonstrated higher accuracy than other models [29]. In addition, in a study by Ichikawa et al. on weight estimation from chest X-ray images, VGG16 accurately estimated over 80% of cases within a 5-kg error margin. Grad-CAM analysis revealed that the model focused on regions such as the diaphragm, neck, and axillary regions [9]. Based on these findings, VGG16 may be more appropriate for tasks that require attention to large anatomical structures or overall balance rather than those necessitating focus on fine structures. Conversely, the high accuracy of VGG16 in our results is likely owing to its ability to focus on the relatively large structures within the images that contributed to determining whether retakes were necessary. A model with fewer layers and a simpler architecture, such as VGG16, may be better suited for extracting these features. In addition, the limited amount of data used in this study may have been well suited for VGG16. Figure 6 shows the considerable differences in the training process and the number of training epochs for each architecture. This is considered to be due to the number of parameters in each model. In addition, the learning curve of VGG16, which had the highest accuracy, showed stable convergence of the loss function for both the training and validation data compared to the other models. The verification of the proposed method also showed that the use of simple convolutional blocks is suitable for transfer learning to chest X-ray images. Tables 1 and 4 show that the assessments were conducted with an accuracy of 80% or higher in 6 of 7 cases where the standard deviation (SD) of the threshold of individual observer was 20% or higher. Therefore, using the proposed RAS method, variation in judgments may reduce among operators regarding the necessity of retaking and help in avoiding unnecessary retakes. Figure 8 indicates that in cases 002 and 016, which were processed with high accuracy, only images with an inspiratory rate near the retaking threshold were incorrectly assessed. On the other hand, in case 021, most images with an inspiratory rate of up to 90% were incorrectly judged as “Retake,” although the retaking threshold was 70%. As shown in Table 1, case 021 had the largest SD of the threshold of individual observer in visual evaluation, indicating large variability in the judgments of the radiological technologist. Therefore, the challenge in this case was considered to be high. This may be due to the fact that compared to other cases, the lateral expansion of the thorax during inhalation was restricted, whereas the vertical expansion was substantial. Table 2 and Fig. 10 show that the CNN focused on the diaphragm and expansion of the thorax, which radiological technologists also pay attention to when checking the inhalation state.

The assessment accuracy of the actual chest X-ray images was 82.3% using VGG16 (Lr = 10^–7^). Precision, F1-score, and AUC also achieved the highest values. The high precision indicated that a large proportion of the images classified as requiring retakes had insufficient inspiration, while the proportion of images with sufficient inspiration incorrectly assessed as “Retake” was low. Therefore, this demonstrates that the implementation of RAS employing VGG16 contributes to reducing unnecessary medical radiation exposure caused by avoidable retakes. Furthermore, the high F1 score and AUC values indicate minimal bias in judgment accuracy and high precision in assessments. Table 6 shows that seven of the ten cases in which the maximal inhalation image was incorrectly assessed as “Retake” were requested by the Department of Pulmonary Medicine. These included cases of interstitial pneumonia, sarcoidosis, and chronic obstructive pulmonary disease. Therefore, although the examinations were completed with maximum inspiration, it is possible that sufficient inspiration was not achieved due to the disease. This is one factor contributing to incorrect assessments. Figure 15 shows that CNN made its assessments by focusing on the ribs, diaphragm, and cardiac shadow, similar to the verification using DDR.

However, generalizing the application of the proposed method remains challenging. First, we only verified the standing frontal image, and no verification was conducted using images taken in different body positions (supine, sitting, or lateral). Although technically feasible, this requires verification once sufficient training data are available. Second, no validation was conducted using the latest CNN architectures, optimization of the CNN structural details, preprocessing of input images (resizing algorithms, matrix size, and alignment), or comparative validation using different data augmentation methods. This is because the primary focus of this study was to demonstrate the potential of deep learning in assessing whether chest X-ray images should be retaken or not. Future studies should include a comparative study of these methods, verification using initial weights pre-trained by medical images for transfer learning and training data that reflect the judgments of the doctors. Finally, the inspiratory rate defined in this study was calculated based on two-dimensional images, and its consistency with the actual intake volume was not verified. In the future, if its reliability is confirmed by comparison with pulmonary function tests, it could be further explored as a regression task for estimating inspiratory rate from chest X-ray images. The estimated inspiratory rate could serve as a reference index for radiological technologists and physicians.

## Conclusion

We developed a RAS for chest X-ray imaging. Verification using DDR and actual chest X-ray images demonstrated a high accuracy of over 80% for both. Implementing the proposed method in hospitals may reduce the variability in judgment among operators and provide doctors with images closer to maximum inspiration, improving their suitability for diagnosis.

## Data Availability

The code generated during the current study is available from the corresponding author on reasonable request. However, the image datasets presented in this study are not publicly available due to ethical reasons.
